# Bioactive compounds, antibacterial and antioxidant activities of methanol extract of *Tamarindus indica* Linn.

**DOI:** 10.1038/s41598-022-13716-x

**Published:** 2022-06-08

**Authors:** Kehinde Oluwakemi Fagbemi, Daniel Ayandiran Aina, Morenike Olutunmbi Adeoye-Isijola, Kubendran Kista Naidoo, Roger Murugas Coopoosamy, Olufunmiso Olusola Olajuyigbe

**Affiliations:** 1 Department of Microbiology, School of Science Technology, Babcock University, PMB 4005, Ilisan Remo, Ogun State Nigeria; 2 Department of Nature Conservation, Mangosuthu University of Technology, Durban, KwaZulu-Natal South Africa

**Keywords:** Drug discovery, Microbiology

## Abstract

*Tamarindus indica* is one of the tropical medicinal plants that has been attributed curative potential of numerous diseases by many rural dwellers. This study was designed to evaluate the antioxidant, antibacterial activities and also to determine the various chemical constituents responsible for its pharmacological activities. The methanol extract of *Tamarindus indica *fruit pulp was analyzed by Gas Chromatography/Mass Spectrometer to determine the volatile compounds present. The antioxidant activities were performed using DPPH and FRAP method and the antibacterial activity was tested against some common pathogens by macro broth dilution method. The GCMS analysis shows the presence of 37 compounds, out of which 14 had their peak area percentages ≥ 1% and only two compounds had no reported pharmacological activities. Most of the bioactive compounds including 5-Hydroxymethylfurfural (31.06%)-3-O-Methyl-d-glucose (16.31%), 1,6-anhydro-β-D-Glucopyranose (9.95%), 5-methyl-Furancarboxaldehyde (3.2%), Triethylenediamine (1.17%), 1-(2-furanyl)-1-Propcanone (2.18%), Methyl 2-furoate (3.14%), Levoglucosenone (3.21%), methyl ester-Hepta-2,4-dienoic acid, (8.85%), 2,3-dihydro-3,5-dihydrox-4H-Pyran-4-one (3.4%), O-α-D-glucopyranosyl-(1.fwdarw.3)-β-D-fructofuranosyl-α-D-Glucopyranoside (2.18%), n-Hexadecanoic acid (1.38%), 2-Heptanol, acetate (1.29%), 5-[(5-methyl-2-fur-2-Furancarboxaldehyde (1.08%), 3-Methyl-2-furoic acid (1.05%) and cis-Vaccenic acid (2.85%)have been reported with different activities such as antibacterial, antifungal, antitubercular, anticancer, antioxidant and other prophylactic activities. The extract demonstrated inhibitory potential against all tested pathogen. However, *Plesiomonas shigellosis* ATCC 15903 and *Bacillus pumillus* ATCC 14884 are more sensitive with the MIC of 0.22 and 0.44 mg/ml respectively. The antioxidant activity was relatively low due to the low phenolic content of the extract. This shows that there is a strong correlation between antioxidant activities and phenolic content. GC–MS analysis revealed the presence of bioactive phytoconstituents with various biological activities and this justifies the rationale behind its usage as a curative therapy by many local dwellers.

## Introduction

Plants play imperative roles in human existence and they are the bedrock of traditional medicine^1^. Unlike the synthetic drugs used for the treatment of various infections, plants are effective, safe, affordable, and with fewer side effects^2^. A larger percentage of these plants are capable of producing numerous categories of secondary metabolites which are the major reason why they are effective for therapeutic purposes even since prehistorical days^3^. Many compounds isolated from these plants have been used as drugs either in their natural form or in semi-synthetic form^4^. While the secondary metabolites are structurally diverse chemical compounds effective against pathogens and environment constraints^5^, these bioactive compounds have been shown to contain great medicinal activities such as antibacterial, antioxidant, antifungal, anti-allergic, anti-inflammatory, antiparasitic, anticancer, and antihypertensive activities^6,7^. They have been utilized for the therapy of mild to chronic ailments such as inflammation, cancer, diabetes, and stomach ulcer^8,9^. Oladeji^10^ reported that 25% of synthetic drugs are produced from plants originally used by orthodox medicine while Welz et al.^11^ indicated that the usage of herbal drugs as complementary or alternate treatment is on the increase globally and many medicines are benefitting greatly from natural products^12^.

*Tamarindus indica* Linn., commonly called Tamarind, is a tropical leguminous evergreen tree, family Fabaceae, subfamily *Caesalpiniaceae*, found throughout Africa and Southern Asia. The plant is made up of about 30–50% pulp, 11–30% shell, and 25–40% seeds^13^. It is one of the plants highly utilized medicinally due to its healing potential in numerous pharmacopeias^14^. The British and American pharmacopeias indicated that the pulp has anti-pyretic, antiscorbutic, purgative, and relief properties for nausea and bile illness^15^. The leaves possess antihelmintic and vermifuge properties destroying intestinal parasites^16^ and are extensively used ethnobotanically in Africa, Asia, and Latin America as antimicrobial and antiseptics^17,18^. The seeds have been used as a therapy for diabetes, fevers, and gastrointestinal infections in traditional settings^19^. The pharmacological activities of the various parts of this plant have been associated with the presence of several phytochemicals such as flavonoids, saponin, alkaloids, tannins, polyphenols, and steroids^20^. While the therapeutic potential of the *T. indica* plant is attributed to the presence of bioactive phytoconstituent available in every part of this plant^21^, its several economic values and health benefits is highly commercialized throughout the world^22^. However, there is a scarcity of information on the bioactive compounds of its methanol extract and its antioxidant and antibacterial activities. Since more awareness has been drawn to the search of novel drugs originating from natural products through innovative technology such as high-throughput selection^23^, the present study investigated the bioactive phytoconstituents available in the methanolic extract of *Tamarindus indica* fruit through the use of GC–MS techniques and indicated the antibacterial and antioxidant activities of the extract.

## Methods

### Sample collection

Mature and dried Tamarind fruits were obtained from the plants growing in its natural habit in Yola, Adamawa state, North East, Nigeria (9.2035° N, 12.4954° E). The fruits were collected in accordance with relevant guidelines and minimum number of fruits required for the accomplishment of the study was collected after permission was taken from the indigenes in whose locality the plant was found. The pulp was removed from the seeds by scrapping with the hand. It was ethno-botanically authenticated by a taxonomist (Dr. Nodaz George) from the University of Lagos herbarium with voucher No. LUH: 8771 and was deposited at the herbarium. Before the analyses, all visible contaminants and infested pulp were removed to ensure healthy and qualitative dried Tamarind fruits.

### Chemical reagents

Only analytical grade chemical reagents and solvents were used for this investigation. They were obtained from Germany, produced by Merck KGaA, with the product name Sigma-Aldrich.

### Extraction of tamarind pulp

The extraction process was carried out by soaking 80 g of scrapped pulp in 640 ml of 70% methanol at room temperature for 72 h and agitated intermittently for proper digestion. Whatman No. 1 filter paper was later used to filter the mixture and the residue was discarded. The solvent was evaporated from the filtrate in a rotary vacuum evaporator (Laborota 4000-efficient, Heidolph city Germany) at 40 °C under pressure until a semisolid concentrate was obtained. The crude extract was allowed to cool down and air-dried at ambient temperature prior to storage in a refrigerator at 4 °C for further use.

### GC–MS analysis

The bioactive phytoconstituents in the extract were analyzed with the aid of Gas Chromatography-Mass Spectrometry (GC–MS) equipment (QP 2010 Plus SHIMADZU)^24^. The GC–MS was equipped with a flame ionization detector. Instrument conditions: injector temperature − 250 °C, detector temperature − 250 °C, oven temperature − 60 °C (Isothermal), flow rate − 2.0 mL/min, split ratio − 10:1, injection volume − 0.5 µL and 24 min run time. The compounds eluted in the methanol extract of *T. indica* were identified by comparing the spectrum of unidentified compounds with those of identified compounds in the NIST MS 2.0 structural library to discover their nomenclatures, molecular weight, and structure^25^.

### Test organisms

Bacteria isolates used in this study included *Escherichia coli* ATCC 8739, *Klebsiella pneumoniae* ATCC 10031, *Pseudomonas aeruginosa* ATCC 19582, *Acinetobacter calcaoceuticus* UP and *Plesiomonas shigelloides* ATCC 15903 for Gram-negative and *Bacillus cereus* ATCC 10702, *Staphylococcus aureus* ATCC 6558, *Bacillus pumilus* ATCC 14884, *Staphylococcus aureus* NCT 6571 and *Staphylococcus aureus* ATCC 6558 for Gram-positive. All bacteria used were collected from the Department of Microbiology, Babcock University, Ilisan Remo, Ogun State Nigeria. All isolates were aseptically introduced into a nutrient broth for resuscitation purposes and incubated at 37 °C for 24 h prior to the antibacterial activity test.

### Preparation and standardization of inoculums

The bacteria isolates were subculture in a broth for a period of 24 h at optimal temperature (37 °C) and a suspension equivalent to a cell density of 1 × 10^8^ CFU/ml was prepared according to McFarland standard for each isolate. Extra dilution was carried out until the cell density reduces to 1 × 10^6^ CFU/ml which was confirmed with the aid of a UV visible spectrophotometer (Thermo electron corporation USA) at an absorbance of 625 nm. The standardization was sustained throughout the experimental period^26^.

### Determination of minimum inhibitory concentration

The minimum inhibitory concentration (MIC) is a technique used to calculate the sample with the least concentration that could inhibit the growth of microorganisms. It was performed using the macro broth dilution method. Prior to the analysis, 1% dimethylsulfoxide (DMSO) was used to dissolve the extract and the concentration varies from 0.1 to 14.04 mg/ml was prepared in the extracting solvent. The reconstituted extract was assessed for sterility by dispensing 1 mL of the extract into 9 mL of sterile nutrient broth before incubating at 37 °C for 24 h. Briefly, the crude extract, the antibiotics (positive control), and saline water (negative control) were serially diluted each in twofold Mueller Hinton broth in the different test tubes to obtain different concentrations of the antibacterial agents. In addition, 100 µl of standardized overnight cultured organisms were inoculated into all the test tubes except the control. The test tubes were incubated for 24 h at 37 °C and observed for any visible growth or turbidity^27^.

### Determination of antioxidant activity

Two different antioxidant assays which include DPPH and FRAP were performed to assess the antioxidant potential of the extracted fruit pulp.

### 2,2-Diphenyl-1-picrylhydrazyl (DPPH) radical scavenging method

Five different concentrations of the extract were thoroughly mixed with 0.2 mM of DPPH prepared with ethanol. The absorbance of each concentration was determined at 517 nm after an incubation period of 30 min in a dark room. The standard used was gallic acid while methanol serves as control^28^. The radical scavenging activity of the extract was calculated by applying the formulae below.

${\text{DPPH scavenging activity }}\left( \% \right) \, = \, \left[ {\left( {{\text{Abs control }}{-}{\text{ Abs sample}}} \right)} \right]/\left( {\text{Abs control}} \right)] \times {1}00;\;$ where; Abs control is the absorbance of DPPH + methanol; Abs sample is the absorbance of DPPH radical + sample (sample or standard).

### Ferric reducing antioxidant power (FRAP)

The FRAP assay focus on the ferric reducing ability of the extract. The reduction of ferric ion (Fe^3+^) to the ferrous ion (Fe^2+^) is visible by the formation of the blue complex (Fe^2+^/TPTZ). Briefly, in the preparation of the working reagent, 100 mL of acetate buffer at 30 mM with 10 mL of a 10 mM TPTZ [2,4,6-tripyridyl-s-triazine] in 40 mM HCL was added to 10 mL of FeCl_3_.6H_2_O at 20 mM. Afterward, 3 ml of the freshly prepared FRAP solution was vigorously mixed with 100 µl of the crude extract (100–500 mg/mL). This resulted in the formation of a blue color complex indicating ferric tripyridyl triazine (Fe^3+^ TPTZ) complex was turned to ferrous (Fe^2+^) ion after 30 min of incubation at 37 °C. The absorbance was then determined at 593 nm. Freshly prepared working solutions of FeSO_4_ were used for calibration^29^. All determinates were indicated as gallic acid equivalents (GAE) in mg per gram dry weight.

### Determination of total phenolics

The total phenolic content (TPC) was evaluated using the Folin-Ciocalteu method. One milliliter of the methanol extract was dropped in a test tube containing 1 ml of Folin-Ciocalteu reagent, whose concentration has been initially reduced to 60% by the addition of water. The mixture was thoroughly mixed by vigorous shaking of the tubes. The introduction of 2 ml 20% (w/v) of sodium carbonate was next and the mixture was left in a dark place for 30 min and absorbance was determined by the use of a UV–Vis spectrophotometer (Jascov-530) at a wavelength of 765 nm. The results obtained were compared with the standardized gallic acid results. The assay results were expressed as milligrams of gallic acid/gram of the dried extract^30^.

### Determination of the total flavonoid content

The total flavonoid content of the methanolic extract was quantified by the aluminum chloride colorimetric assay for quantitative determination. Briefly, 1 ml of the extract was added to 2.8 mL of distilled water and mixed with 0.1 mL of 1 mg/mL potassium acetate solution. Then, 0.1 mL of 10% aluminum chloride was added to this solution. After 30 min of incubation, the absorbance was monitored and measured at 415 nm by using a UV–visible spectrophotometer^31^. Total flavonoid content was expressed as gallic acid equivalents (GAE) in milligram/gram dry weight.

### Statistical analysis

All the antioxidant analyses, total phenolic, and flavonoid content were performed in triplicate, and average values and their standard derivations of the results were presented. The relationship among DPPH, FRAP assay, TPC, and TFC was evaluated by the use of correlation.

## Results

**Figure 1:**
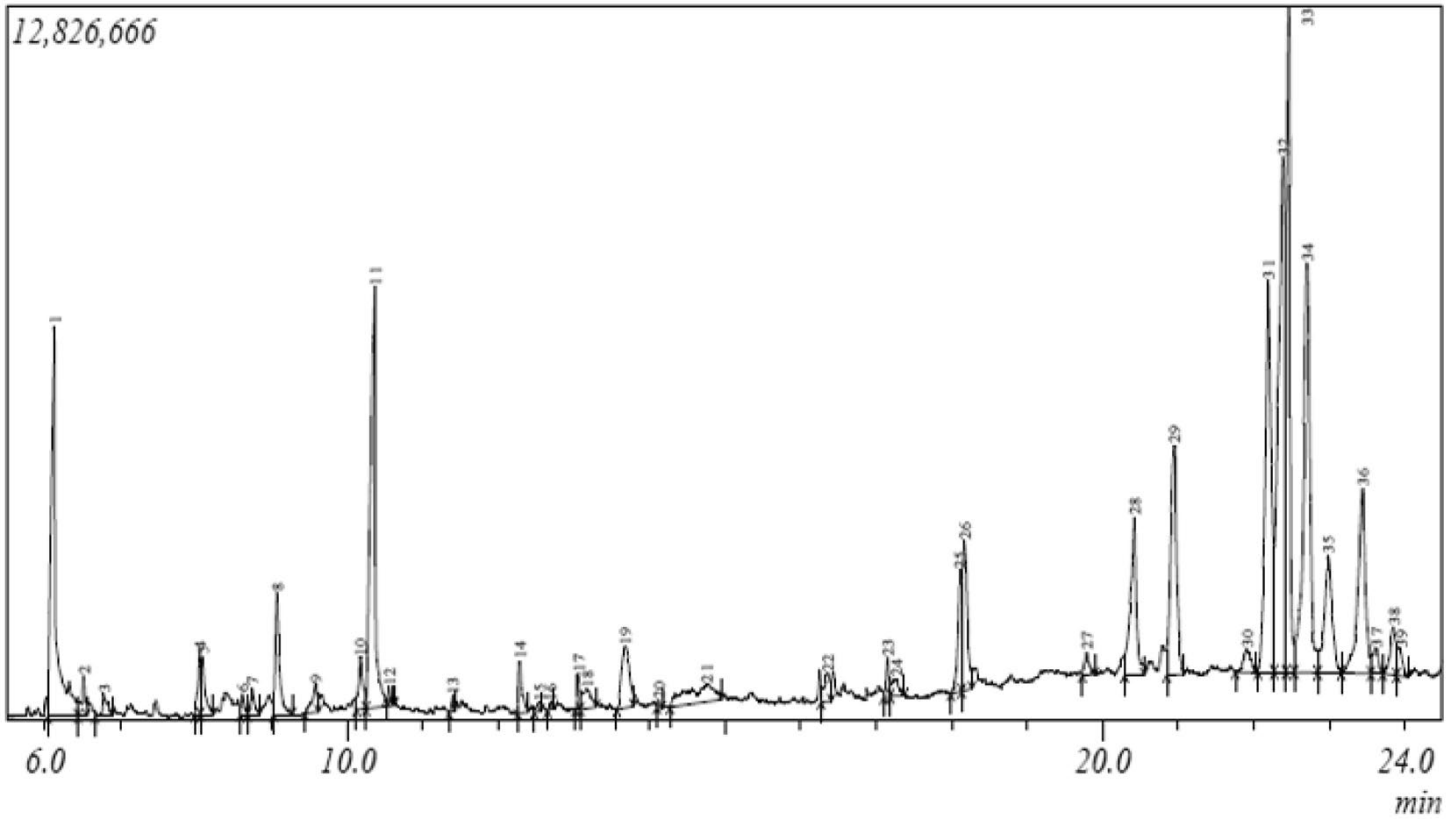
GC–MS Chromatogram of the phytoconstituents present in the methanol extract of *Tamarindus indica.*

Gas chromatography-mass spectrometry (GC–MS) was used to analyze the methanol extract to identify the bioactive compounds present in the fraction. The GC–MS chromatogram of these bioactive compounds is presented in Fig. 1 and their peak area percent, chemical structure as well as biological activities are represented in Table 1. From the GC–MS analysis, 37 bioactive compounds were identified and expressed as percentages of the peak area relative to the total peak area. Bioactive compounds with greater than or equal to 1.0% peak areas were identified as the prominent bioactive compounds. The percentages of bioactive compounds > 1% are 5-Hydroxymethylfurfural (31.06%), -3-O-Methyl-d-glucose (16.31%), 1,6-anhydro-β-D-Glucopyranose (9.95%), 5-methyl-Furancarboxaldehyde (3.2%), Triethylenediamine (1.17%), 1-(2-furanyl)-1-Propcanone (2.18%), Methyl 2-furoate (3.14%), Levoglucosenone (3.21%), methyl ester-Hepta-2,4-dienoic acid, (8.85%), 2,3-dihydro-3,5-dihydroxy-4H-Pyran-4-one (3.4%), O-α-D-glucopyranosyl-(1.fwdarw.3)-β-D-fructofuranosyl-α-D-Glucopyranoside (2.18%), n-Hexadecanoic acid (1.38%), 2-Heptanol, acetate (1.29%), 5-[(5-methyl-2-fur-2-Furancarboxaldehyde (1.08%), 3-Methyl-2-furoic acid (1.05%) and cis-Vaccenic acid (2.85%). The pharmacological activities of Hydroxymethylfurfural with the highest peak area percentage and those of other bioactive compounds have been reported as being of therapeutic importance while those of 2-ethyl-2-Butenal (0.3%) and 3-(hydroxymethyl)-6-2-Cyclohexene-1-one (0.42%) with lower peak area percentages, having no reported pharmacological activities, were as shown in Table 1.

**Table 1:** Showing the names, chemical structure, molar mass, molecular formulae, and the biological activities of each compound identified by GCMS analysis.

Peak numbers	R. time	Peak area %	Height %	A/H	Mark name	Chemical formulae	Molecular structure	Molecular weight (g/mol)	Biological activities
1	5.727	0.14	0.32	4.28	1-(2-furanyl)-Ethanone	C_6_H_6_O_2_		110	Antioxidant and antifungal agent^32^
2	5.932	0.4	1.54	2.62	2(5H)-Furanone	C_4_H_4_O_2_		84	Antimicrobial agent^33^
3	6.043	0.3	0.88	3.46	2-ethyl-2-Butenal	C_6_H_10_O		98	Not reported
4	6.21	0.75	2.31	3.27	5-methyl-2(5H)-Furanone	C_5_H_6_O_2_		98	Flavoring agent, antifungal and antibacterial^34^
5	6.251	0.27	1.42	1.9	N-cyclohexylidene-Methanamine,	C_7_H_13_N		111	Anticonvulsant agent^35^
6	6.379	3.2	8.17	3.93	5-methyl-2-Furancarboxaldehyde	C_6_H_6_O_2_		110	Fungicide and nematicide, antibacterial, antiinflammatory, Proteinase inhibitor^36^
7	6.451	0.57	1.89	3.02	1,4,5-trimethyl-imidazole	C_6_H_10_N_2_		110	Carcinogenic^37^
8	6.584	0.57	1.72	3.36	1-(3-thienyl)-ethanone	C_6_H_6_OS		126	Antifungal, antitubercular, and anticancer activities^38^
9	6.653	0.87	4.37	1.99	2,4-Dihydroxy-2,5-dimethyl-3(2H)-furan-3	C_6_H_8_O_4_		144	Antibacterial and antifungal activities Flavoring^39^
10	6.69	0.42	1.79	2.35	4-oxo-methyl ester-pentanoic acid	C_6_H_10_O_3_		130	Antioxidant and antimicrobial agent^40^
11	6.957	1.17	1.41	8.31	Triethylenediamine	C_6_H_12_N_2_		112	Herbicidal and antibacterial^41^
12	7.483	0.53	1.77	3	Benzeneacetaldehyde	C_8_H_8_O		120	Antioxidant, mutagenic, and antimicrobial^42^
13	8.194	2.18	3.97	5.52	1-(2-furanyl)-1-propanone	C_7_H_8_O_2_		124	Antibacterial^43^
14	8.272	3.14	4.13	7.63	Methyl 2-furoate	C_6_H_6_O_3_		126	Biofilm inhibitors, Antifungal, Antioxidant activity^44^
15	8.723	3.21	3.78	8.52	Levoglucosenone	C_6_H_6_O_3_		126	Anticancer and antitumor activity^45^
16	9.281	8.85	11.32	7.84	methyl ester-Hepta-2,4-dienoic acid	C_8_H_12_O_2_		140	Antioxidant activity^46^
17	9.417	3.4	3.19	10.69	2,3-dihydro-3,5-dihydroxy-4H-pyran-4-one	C_5_H_6_O_4_		130	Antibiofilm, Melanin production inhibitor, Antioxidant, Antimicrobial activity^47^
18	10.302	1.05	1.64	6.39	3-Methyl-2-furoic acid	C_6_H_6_O_3_		126	Bactericidal, fungicidal and nematocidal agent^48^
19	11.175	31.06	8.74	35.66	5-Hydroxymethylfurfural	C_6_H_6_0_3_		126	Antioxidant, antimicrobial, Antiproliferative, Antibiofilm^49^
20	11.388	0.42	0.69	6.16	3-(hydroxymethyl)-6–2-C cyclohexen-1-one	C_7_H_10_O_2_		126	Not reported
21	11.952	1.29	1.61	8.05	2-Heptanol, acetate	C_9_H_18_O_2_		158	Ovicidal and lavicidal^50^
22	12.406	0.47	0.53	8.9	O-α-D-glucopyranosyl-(1.fwdarw.3)-β-D-fructofuranosyl-α-D-Glucopyranoside	C_18_H_32_O_16_		504	Anti-diabetic, anti-hyperlipidemic, anti-oxidant activity^51^
23	12.838	0.47	1.11	4.25	5-(2-furanylmethyl 2-furancarboxaldehyde	C_10_H_8_O_3_		176	Antimicrobial^52^
24	13.612	0.3	0.75	4.01	3,5-Dimethyl-1H-pyrazol-4-yl)acetic acid	C_7_H_11_N_3_O_2_		154	Herbicidal and antimicrobial^53^
25	13.744	1.08	3.68	2.95	5-[(5-methyl-2-furanyl)-methyl2-furancarboxaldehyde	C_6_H_6_O_2_		190	fungicide and nematicide^54^
26	14.803	9.95	2.91	34.25	1,6-anhydro-β-D-glucopyranose	C_6_H_10_O_5_		162	Human metabolites, biomarkers, anti-human immunodeficiency virus, and blood anti-coagulant^55^
27	15.573	2.18	1.52	14.34	O-α-D-glucopyranosyl-(1.fwdarw.3)-β-D-fructofuranosyl-α-D-glucopyranoside	C_18_H_32_O_16_		504	Anticonvulsant, antioxidant, antitumor, antibactetria^56^
28	16.885	16.31	5.43	30.12	3-O-Methyl-d-glucose	C_7_H_14_O_6_		194	Preservatives, anti-inflammatory, and antitumor agent^57,58^
29	17.21	1.38	5.19	2.68	n-Hexadecanoic acid	C_16_H_32_O_2_		256	Nematicide, Hemolytic, Anti-androgenic, Antidiabetic Hypocholesterolemic, Anti-oxidant and pesticidal^59^
30	17.508	0.28	0.97	2.89	9-oxabicyclo[6.1.0]non-6-en-2-one	C_8_H_10_O_2_		138	Unknown
31	17.702	0.32	0.48	4.54	2-(hydroxymethyl)-cyclohexanone	C_7_H_12_O_2_		128	Antibacterial activities^60^
32	18.052	0.38	2.19	1.76	methyl ester, (Z)-7-hexadecenoic acid	C_17_H_32_O_2_		268	Antimicrobial and antioxidant^61^
33	18.368	2.35	6.68	3.53	cis-Vaccenic acid	C_18_H_34_O_2_		283	Antibacterial and hypolipidemic activity^62^
34	18.459	0.09	0.67	1.31	Octadecanoic acid	C_18_H_36_O_2_		285	Anticancer and antibacterial activity^63^
35	20.534	0.11	0.48	2.17	2-hydroxy-1-(hydroxymethyl)ethyl ester-hexadecanoic acids	C_19_H_38_O_4_		330	Antioxidant, antimicrobial, Flavoring agent, 5-Alpha reductase-inhibitor, Pesticide, Antifibrinolytic, and Hemolytic agent^64^
36	21.796	0.55	0.52	10.61	(Z)-2,3-dihydroxypro-9-octadecenoic acid	C_21_H_40_O_4_		357	Flavoring agent, Lubricant, Antioxidant, Larvicidal, and analgesic activities^65^
37	22.229	0.12	0.22	5.45	β-Sitosterol	C_29_H_50_O		414	Antimicrobial, Antioxidant anti-inflammatory, antidiabetic, Immunomodulatory, Anthelminthic and Anti-mutagenic Activities^66^

The methanol extract of *T. indica Linn* fruit pulp was evaluated for its antimicrobial potential against the test isolates using the macro tube dilution method. The ability of this extract to prevent the growth of the tested organisms was in Table 2. The result displayed the ability of the extract to suppress the growth of the bacterial isolates at varying concentrations. *P. shigelloides* ATCC 15903 was the most susceptible at MIC as low as 0.22 mg/mL while *B. cereus* ATCC 10702 and *K. pneumoniae* ATCC 10031 had the same MIC of 3.51 mg/mL. *A. calcoaceuticus* UP, *S. aureus* ATTC 6558, and *S. aureus* NCTC 6571 showed some level of resistance with MIC greater than 7.02 mg/mL though the minimum inhibition concentrations of the bacterial isolates varied from 0.0195 and 1.25 µg/ml for ciprofloxacin used as control.

**Table 2:** MIC of *T. indica* extract against tested bacterial isolates.

	Methanol extract	Ciprofloxacin
Test bacterial isolates	MIC (mg/ml)	(µg/ml)
*E. coli* ATCC 8739	1.76	0.0195
*B. cereus* ATCC 10702	3.51	0.0781
*K. pneumoniae* ATCC 10031	3.51	0.0195
*P. aeruginosa* ATCC 19582	0.88	0.0195
*S. aureus* ATCC 6558	7.02	0.0391
*A. calcoaceuticus* UP	7.02	1.25
*P. shigelloides* ATCC 15903	0.22	0.0391
*B. pumilus* ATCC 14884	0.44	0.0195
*S. aureus* NCTC 6571	7.02	0.0391

**Figure 2:**
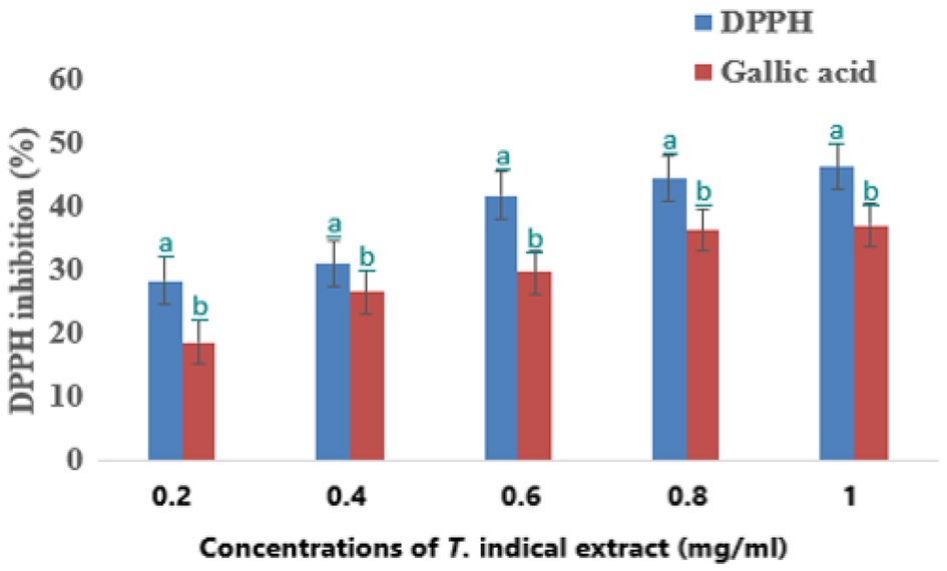
DPPH radical scavenging activity (%) (Mean + Standard deviation) of *T. indica* fruits extract.

The IC_50_ values of the extract for each antioxidant parameter assayed are 5.34, 18.67, and 7.34 for DPPH, FRAP, and Gallic acid, respectively. In the DPPH assay, the extract exhibited a concentration-dependent radical scavenging activity and this activity increases significantly with increase concentration. The results obtained from different concentrations are compared to the Gallic acid (ρ < 0.05) as shown in Fig. 2.

**Figure 3:**
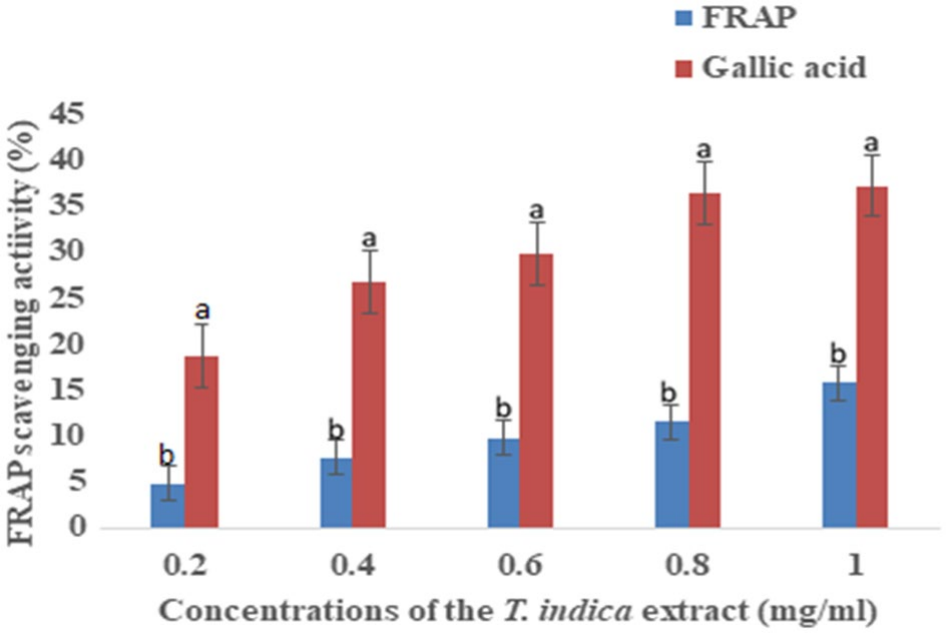
FRAP activity (%) (Mean + Standard deviation) of the extract of *T. indica* fruits.

FRAP analysis was carried out to ascertain the antioxidant properties of the methanolic extract focusing on its potential to reduce ferric (III) to ferrous (II). The mean scavenging activities with different superscripts at the same concentration of the extract are notably different (ρ < 0.05) as shown in Fig. 3. The assessment of FRAP activity using gallic acid was remarkably higher than those of the extract from the *T. indica* fruit.

**Figure 4:**
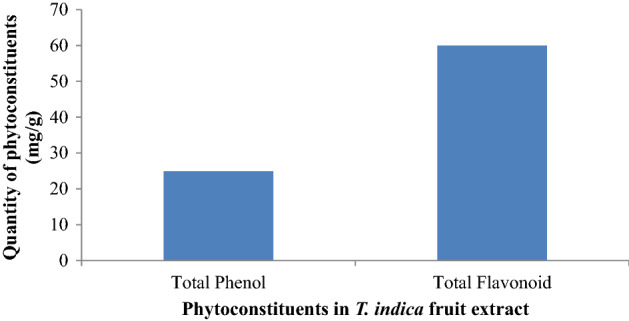
Total phenolic and flavonoid contents of methanol extract of *T. indica* fruit.

The gross phenolic and flavonoid content of the methanol extract was also assessed and compared with that of the standard (Gallic acid). The overall quantity of the flavonoid content of the fruit extract was higher than the phenolic content as shown in Fig. 4.

## Discussion

The global increase in demand for plant-derived products for therapeutic and nutraceutical purposes have stimulated the quest to identify the chemical compounds present in each plant and their various pharmacological activities. In addition, the need for researchers to search for safer antioxidants from natural sources over synthetic ones such as BHT, BHA, propyl gallate, and tertbutyl-hydro quinine which are known to be carcinogenic has increased over the years as well^67^. Thus, the consumption of natural products such as fruits and vegetables showing strong antioxidant activities in preventing heart diseases and several cancerous ailments becomes necessary^68^. The GCMS analysis showed that the pulp has potential novel compounds that could be isolated for therapeutic purposes and irrespective of the percentages of the identified compounds, scientific reports showed that each of the compounds possessed significant therapeutic potentials.

Although the pharmacological activities of the major and minor bioactive compounds of plants are rarely reported and pharmacological activities of plants are mostly attributed to the flavonoids, alkaloids and phenolic compounds, Shapla et al*.*^69^ indicated that 5-Hydroxymethylfurfural (HMF) is an organic compound that possesses several beneficial potentials including antioxidant, anti-allergic, antiproliferative, anti-sickling, anti-hypoxic and anti-hyperuricemic impacts while Rajkumari et al*.*^49^ documented its antibiofilm activity. Its mechanism of antimicrobial action was related to growth or proliferation inhibition. Similarly, these inhibitory activities of 5-Hydroxymethylfurfural were reported by Palchykov et al*.*^70^ as the major compound of *Punica granatum* peel extract with deleterious effect on bacteria, protists, and nematodes. This is in agreement with an earlier study by Ahmed and Ayoub^71^. Their studies affirmed 5-Hydroxymethylfurfural as one of the main compounds in *Tamarindus indica* pulp extract with over 30% of the extract component. While 3-O-Methyl-d-glucose (16.31%), identified also as a component of the *Tamarindus indica* pulp extract, have been implicated in preservative activities as well as antitumor and anti-inflammatory potentials^57,58^, other bioactive components of the extract with their various pharmacological activities have been reported in many studies as bactericidal, fungicidal, nematocidal and antioxidant agents^48^. Supaphon and Preedanon^56^ also reported on the anti-convulsant, antioxidant, antitumor, and the anti-bacterial potential of O-α-D-glucopyranosyl-(1.fwdarw.3)-β-D-fructofuranosylα-D-Glucopyranoside. That 2-ethyl-2-Butenal and 3-(hydroxymethyl)-6-2-Cyclohexen-1-one had no pharmacological activities reported from the literature search could mean that these are new compounds that may not have been previously identified in medicinal plants.

Furthermore, in this study, the test organisms could have been inhibited by this extract due to the presence of β-sitosterol, cis-Vaccenic acid and other compounds earlier reported to have antimicrobial potentials though their real mode of action on microorganisms is not clearly understood. The minimum inhibitory concentration of the extract against *E. coli* ATCC 8739 (1.75 mg/ml), *Pseudomonas aeruginosa* ATCC 19582 (0.88 mg/ml) and *P. shigelloides* ATCC 15903 (0.22 mg/ml) showed the extract has stronger antimicrobial activities against the Gram-negative bacteria. This supports the findings of Abukakar et al.^72^, Adeola et al.^73^, and Bhadoriya et al.^74^ that showed that *T. indica* extract was capable of suppressing the growth of the test organisms. This implies that the extract would be an effective therapy for infections such as wounds, dysentery, diarrhea, and food poisoning in which the test organisms have been implicated^75–77^.

Since some metabolic and age-related ailments are intimately linked with oxidative activities, therefore the exploitation of herbs and spices as a natural origin of antioxidants to prevent oxidation deserves more awareness^78^. While Luengthanaphol et al.^79^ reported that ethanol extract of tamarind seed coat exhibited antioxidant activity, this study indicated that methanol extract of the pulp has strong antioxidant properties. Although a good correlation has been recognized between antioxidant capacity and ferric reducing potential of the extract, the radical scavenging and ferric reducing potentials were relatively low when compared with that of the standard. This is in agreement with Atawodi et al.^80^ and Reis et al.^81^ indicating that extracts of *T. indica* displayed high antioxidant activities and Ugwuona and Onweluzo^82^ reported that Tamarind pulp possesses high antioxidant activities at elevated extraction temperature. Many reports have shown strong interdependence between antioxidant activities, phenolic and flavonoid content of plant extracts^83,84^, a strong positive correlation was also noticed between the total phenolic (r = 0.9912, *p* ˃ 0.05) and antioxidant activities evaluated by DPPH (r = 0.8938, *p* ˂ 0.05) and FRAP (r = 0.9808, *p *˂ 0.05) assays.

## Conclusion

In conclusion, the bioactive compounds, antibacterial and antioxidant activities of methanol extract of *T. indica* fruit pulp were investigated in vitro and the various pharmacological activities of each bioactive compound in this extract were identified. The pulp extract showed effective antibacterial and antioxidant activities while the pharmacological activities of the extract could be attributed to the bioactive compounds identified in the pulp extract and justify more reasons for the numerous usages of the plant in ethnomedicine.

## Data Availability

All data generated or analysed during this study are included in this published article.
